# Early diagnostic challenges of isolated ocular motor nerve palsy: diabetic, ischemic, or Tolosa-Hunt Syndrome

**DOI:** 10.3389/fneur.2025.1592993

**Published:** 2025-07-17

**Authors:** Ling Yu, Wei Qin, Wenli Hu, Lei Yang

**Affiliations:** ^1^Department of Endocrinology, Beijing Chaoyang Hospital, Capital Medical University, Beijing, China; ^2^Department of Neurology, Beijing Chaoyang Hospital, Capital Medical University, Beijing, China

**Keywords:** ophthalmoplegia, painful ophthalmoplegia, Tolosa-Hunt Syndrome, microvascular ocular motor nerve palsies, diabetes, small vessel disease

## Abstract

**Objective:**

Investigating the clinical features and etiological diagnosis of early isolated ocular motor nerve palsy to deepen understanding of the condition.

**Methods:**

We retrospectively enrolled 68 patients with isolated ocular motor nerve palsy admitted our hospital between 2017 and 2024. A retrospective analysis was conducted to assess their clinical and imaging characteristics. Based on current diagnostic criteria, patients were categorized into one of the following groups: diabetic ophthalmoplegia (DO), microvascular ocular motor nerve palsies (MVP), or Tolosa-Hunt syndrome (THS). Patients were divided into two groups based on the presence of diabetes, and the clinical and imaging differences between the two groups were compared.

**Results:**

Of the 68 patients, 40 were male, with an average age of 61 years. There were 43 patients with diabetes, and 40 had a history of hypertension. The number of patients with isolated 3rd, 4th, and 6th nerve palsy was 42, 15, and 11, respectively. Sixty patients experienced headache or orbital pain. 46 patients met the criteria for MVP. Among them, 31 patients had DO, and 15 non-diabetic patients also met the criteria for MVP. Of the 46 patients, 22 showed abnormalities on contrast-enhanced MRI. 19 patients were diagnosed with THS. In the diabetic and non-diabetic groups, 11 and 9 patients, respectively, were diagnosed with THS. The number of patients receiving steroid treatment in the diabetic and non-diabetic groups was 38 and 23, respectively, with pain relief rates within 3 days of 70 and 56%, *p* > 0.05.

**Conclusion:**

Currently, the boundaries between DO, MVP, diabetes combined with THS, and benign THS remain unclear. There is a need for clinical research involving specialists in neurology, ophthalmology, and otolaryngology to establish standardized definitions, classifications, and diagnostic criteria.

## Introduction

Patients with diplopia caused by ocular motor nerve palsies often present to ophthalmology, neurology, or neurosurgery departments. The etiologies of ocular motor nerve palsies are diverse, including vascular, inflammatory, infectious, autoimmune, compressive, traumatic, and other causes. Due to limited awareness of the condition, patients frequently seek initial care in ophthalmology or emergency departments, after which they may be referred to neurology, neurosurgery, otolaryngology, or endocrinology. Given its multi-disciplinary nature, clinicians must remain vigilant to prevent misdiagnosis.

Current approaches to the diagnosis and treatment of ocular muscle palsy remain uncertain and controversial (1–3). The differential diagnosis between isolated ocular motor nerve palsy (IOMP) and multiple ocular motor nerve palsies varies in terms of etiology (4). Isolated ocular motor nerve palsy has a relatively narrower range of causes. Various types of ocular motor nerve palsy, including painful ophthalmoplegia (PO), microvascular ocular motor nerve palsies (MVP), and diabetic ocular muscle palsy (DO), are associated with orbital pain or headache. The question arises whether diabetes should be considered a risk factor for MVP or if DO should be regarded as a separate entity. How should DO, MVP, and Tolosa-Hunt Syndrome (THS) be differentiated? Some practitioners even equate THS with PO.

For patients with IOMP, the most accessible clinical information at first contact includes whether they have accompanying headache or orbital pain, a history of diabetes, and previous vascular risk factors. In diabetic patients, peripheral neuropathy may initially be considered, or a diagnosis of MVP may be proposed. If patients present with pain symptoms, THS may be considered.

How should a diagnosis be made when a patient presented with pain symptoms and a history of diabetes? If MRI of the brain and cavernous sinus did not clearly reveal pathology, the diagnosis can be challenging for clinicians.

This paper focused on patients with IOMP and summarized the clinical characteristics, diagnosis, and treatment of IOMP patients admitted to our institution in recent years. We reviewed the literature and provided clinical management recommendations to improve clinicians’ understanding of ocular muscle palsy.

## Materials and methods

### Study subjects and methods

Patients with ocular muscle palsy admitted to the Department of Neurology at our hospital from March 2017 to July 2024 were identified through the hospital information system. The inclusion criteria were as follows: (1) Patients presenting with isolated third, fourth, or sixth cranial nerve palsy within 30 days of onset; (2) Patients who underwent head MRI examination. The research was conducted in line with the Declaration of Helsinki and adhered to the STROBE guidelines (5). Beijing Chaoyang Hospital’s institutional ethics committees granted ethical approval for this study. Informed consent was obtained from all patients prior to the imaging examinations, and all identifiable personal information was anonymized to protect patient privacy.

Exclusion criteria: (1) Patients with ophthalmoplegia due to other causes such as brainstem infarction, myasthenia gravis, aneurysms, pituitary tumors, infections, or trauma were excluded. (2) Patients with paralysis of multiple ocular motor nerves and those with a history of tumors were also excluded. (3) Patients with incomplete clinical data were excluded.

A standardized form was used to collect patients’ general demographic data, vascular risk factors, as well as clinical, laboratory, and imaging data. Vascular risk factors included hypertension, diabetes, cerebral infarction, and coronary atherosclerotic heart disease. Key clinical data included the presence and duration of preceding headache and orbital pain, the affected ocular motor nerve, pupil involvement, use of steroids, and time to symptom improvement. Laboratory tests included blood glucose, glycated hemoglobin, complete blood count, liver and kidney function, among others, with a minority of patients undergoing lumbar puncture for cerebrospinal fluid analysis. All patients received ophthalmology consultations during hospitalization to confirm the involvement of the ocular motor nerve. All patients underwent head MRI, and all received either head MRA or head and neck CTA. Some patients also underwent enhanced MRI of the cavernous sinus or orbit.

According to medical records, third and sixth cranial nerve palsies were easily identified. However, trochlear nerve (fourth cranial nerve) palsy was more difficult to diagnose. If a patient presented with vertical diplopia, head tilt to the side opposite the symptoms, and relatively normal eye movement, it was considered indicative of trochlear nerve palsy.

For patients presenting with ophthalmoplegia accompanied by ipsilateral headache and/or eye pain at their initial visit, regardless of the presence of diabetes, we made a provisional diagnosis of painful ophthalmoplegia. Further laboratory and imaging studies were conducted to differentiate whether the condition was MVP or THS.

Painful ophthalmoplegia is a syndrome with various etiologies. It is defined as a combination of orbital or unilateral headache, ipsilateral oculomotor nerve palsy, sympathetic nerve palsy (Horner syndrome), or sensory loss in the ophthalmic branch of the trigeminal nerve, and occasionally in the maxillary branch distribution.

The definition of THS follows the third edition of the International Classification of Headache Disorders and includes the following criteria: unilateral headache, meeting two of the following: (i) Headache occurs around the ipsilateral eyebrow and orbit. (ii) Headache appears within 2 weeks before, or simultaneously with, palsy of the III, IV, and/or VI cranial nerves. Ipsilateral palsy of one or more of the III, IV, and/or VI cranial nerves, with MRI or biopsy showing granulomatous inflammation in the cavernous sinus, superior orbital fissure, or orbit. The condition cannot be better explained by another diagnosis.

MVP is defined as the acute onset of diplopia and ophthalmoplegia caused by involvement of a single ocular motor nerve (III, IV, or VI), which can spontaneously improve gradually to normal or near-normal levels. The main risk factors include diabetes, hypertension, hyperlipidemia, coronary atherosclerotic heart disease, and smoking or alcohol abuse. Diabetic Ophthalmoplegia is defined as the occurrence of diplopia in diabetic patients due to involvement of one or more ocular motor nerves (III, IV, or VI). When other causes have been excluded, it is considered to be diabetic ophthalmoplegia.

The imaging data of the patients were independently analyzed by two neurological experts. The main focus was to identify abnormalities in the cavernous sinus and orbit, such as asymmetrical signals in the bilateral cavernous sinus and orbital apex, and abnormal enhancement involving the adjacent meninges. Additionally, concurrent sinusitis and stenosis of the internal carotid artery in the cavernous segment were also recorded.

The patients were divided into two groups based on the presence of diabetes. The first group consisted of patients with diabetes, while the second group included those without diabetes. We compared the two groups in terms of the involvement of cranial nerves, presence of pain, risk factors for cardiovascular and cerebrovascular diseases, initial suspicion of THS, positive contrast-enhanced MRI findings, presence of sinusitis, concurrent ipsilateral internal carotid artery siphon stenosis, and the proportion of patients who showed improvement within 3 days of steroid therapy.

Statistical analysis was performed using SPSS 20.0 software. Continuous variables with a normal distribution were expressed as mean ± standard deviation, while non-normally distributed data were expressed as M (P25, P75) and analyzed using the independent samples rank-sum test. Categorical variables were compared using the χ^2^ test. Statistical significance was set at *p* < 0.05, using SPSS 20.0 for analysis.

## Results

During the study period, a total of 98 patients with ocular motor nerve palsy were identified. Thirty patients with potential secondary causes of ocular muscle palsy were excluded. These included 8 cases of multiple cranial nerve palsies, 7 cases with a history of tumors, 2 cases with pituitary adenomas, 2 cases with herpes zoster virus infections, 1 case diagnosed with viral meningitis, 1 case of uremia, and 1 case with ankylosing spondylitis. Additionally, 8 patients did not complete cerebrovascular evaluations.

The study ultimately included 68 patients, of whom 40 were male and 28 were female, with an average age of 61 ± 11 years. Among them, 43 patients had diabetes, including 4 newly diagnosed cases. The duration of diabetes ranged from 1 to 30 years. Patients with a history of hypertension, coronary atherosclerotic heart disease, and stroke were 40, 12, and 10, respectively. Isolated third nerve palsy was observed in the majority of patients (42 cases, 61.8%), with 13 of them showing pupil involvement. Fourth and sixth nerve palsy were found in 15 and 11 patients, respectively. Most patients (60 cases) presented with headache or orbital pain, with a median duration of premonitory pain of 1 day. (Table 1).

**Table 1:** Clinical characteristics of mononeuritis ophthalmoplegia.

Clinical features	Total (*n* = 68)
Age (years)	61.3 ± 11.3
Male	40 (58.8%)
Diabetes	43 (63.2%)
Hypertension	40 (58.8%)
History of CAD	12 (17.6%)
History of stroke	10 (14.7%)
Nerve involved	
CN III	42 (61.8%)
CN IV	15 (22.1%)
CN VI	11 (16.2%)
Headache or orbital pain	60 (88.2%)
Duration of headache *[M(P25, P75)]*	1 (1–2)
Presumed patient with PO (%)	60 (88.2)
Positive head MRI (%)	2 (2.9%)
Complete enhanced MRI (%)	46 (67.6%)
Positive enhanced MRI (%)	22/46 (47.8%)
Stenosis of ICA siphon (%)	29 (42.6%)
Sinusitis (%)	46 (67.6%)
Fasting blood glucose	7.15 ± 3.03
HbA1c (%)	7.2 ± 1.9
Hormonal therapy (%)	61 (89.7%)
Improvement in pain within 3 days (%)	44 (64.7%)
Duration of hospital stay	10.2 ± 3.2

**Figure 1:**
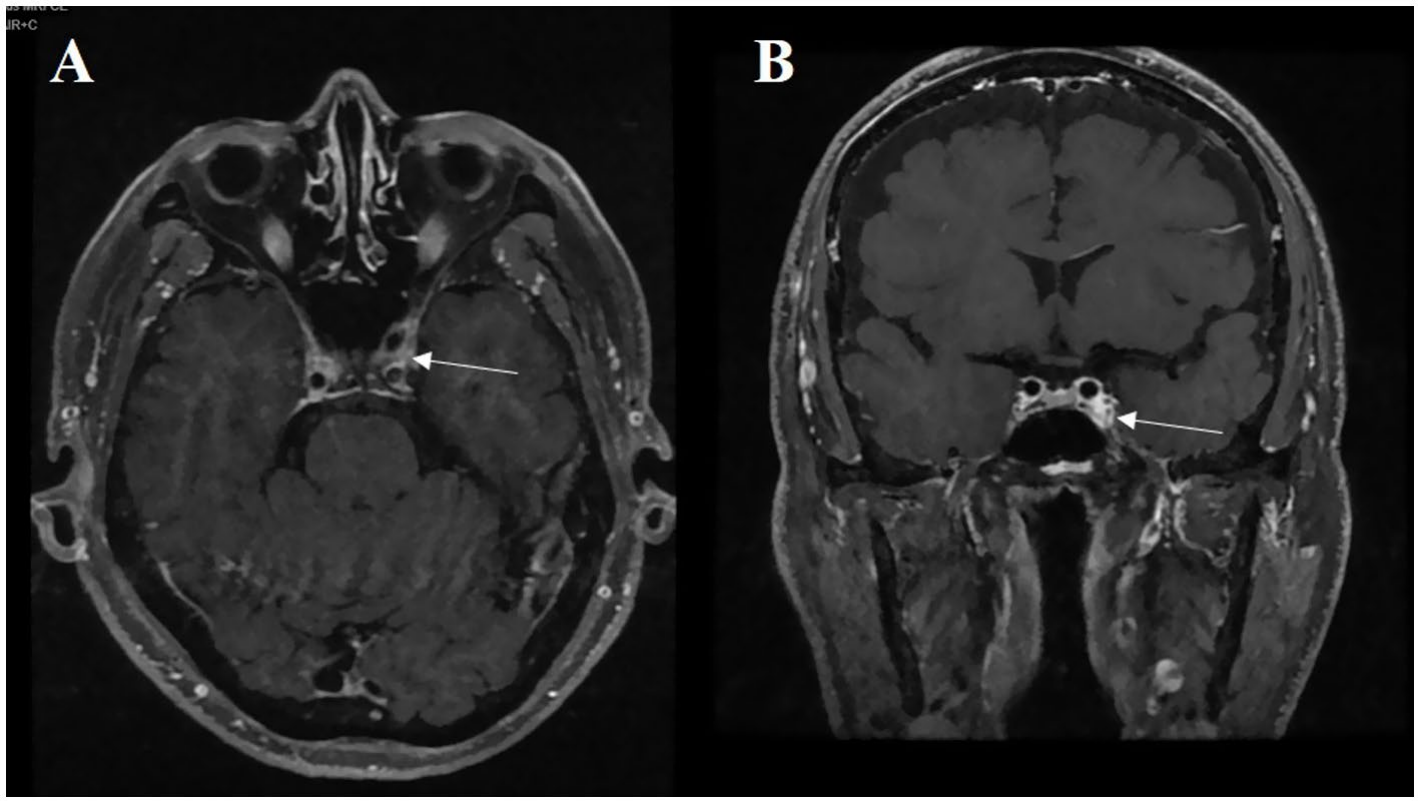
A 74-year-old male patient presenting with vertical diplopia. **(A)** The abnormal asymmetric high intensity was found in the cavernous sinus on the enhanced axial T1-weighted image (white arrow). **(B)** On the enhanced coronal T1-weighted image, we can observe the same abnormal phenomenon (white arrow).

**Figure 2:**
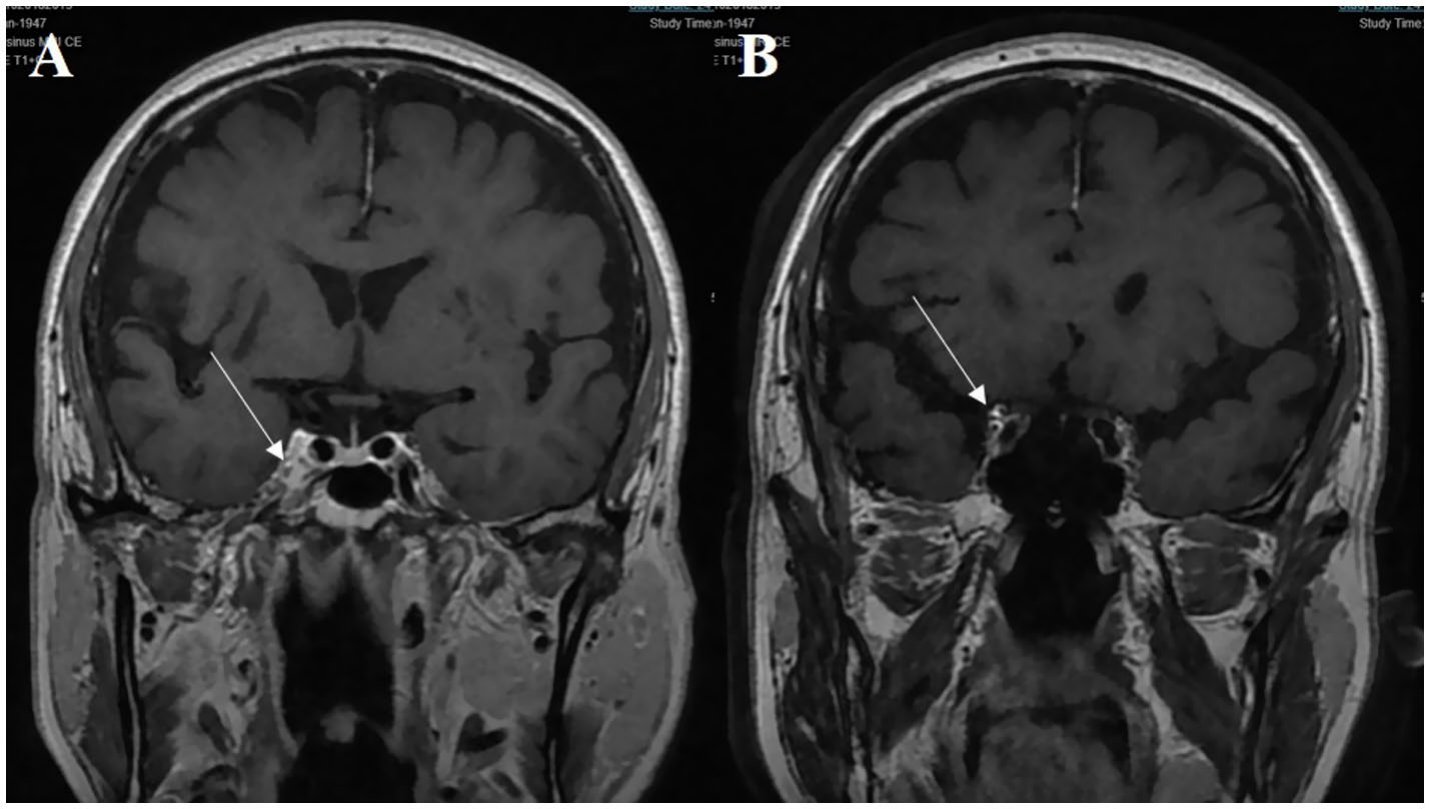
A 70-year-old male patient presenting with third cranial nerve palsy. **(A)** The abnormal asymmetric high intensity was found in the cavernous sinus on the post-gadolinium coronal T1-weighted image (white arrow). **(B)** The lesion extends toward the ipsilateral orbital apex on the coronal enhanced T1-weighted image (white arrow).

At the initial visit, 60 patients were provisionally diagnosed with painful ophthalmoplegia. A total of 46 patients underwent enhanced MRI scans of the cavernous sinus and/or orbit, with positive findings in 22 cases (Figures 1, 2). Among these, one patient had a prodromal headache lasting 25 days, and two patients had no significant pain. A total of 19 patients were ultimately diagnosed with THS. Routine head MRI scans revealed positive results in only 2 patients. Based on the results of the routine head MRI, all 60 patients could be provisionally diagnosed with THS. Sixty-one patients received steroid treatment at varying doses, with a pain improvement rate of 64.7% within 3 days.

According to the diagnostic criteria for painful ophthalmoplegia, 60 patients met this standard. Based on the definition of MVP, 46 patients fulfilled the criteria. Among the diabetic patients, after excluding 12 cases with abnormalities on contrast-enhanced MRI, 31 were diagnosed with diabetic ophthalmoplegia. These 31 patients also met the diagnostic criteria for MVP. Additionally, 15 non-diabetic patients met the MVP criteria.

Patients were divided into two groups based on whether they had diabetes. Aside from glucose-related indices, significant differences between the two groups were observed only in gender, history of coronary artery disease, and incidence of sinusitis. Both the diabetic and non-diabetic groups had only one positive result on non-contrast head MRI scans. Following additional contrast-enhanced MRI of the cavernous sinus and/or orbit, 11 patients in the diabetic group and 9 in the non-diabetic group were clinically diagnosed with THS. A total of 38 diabetic patients and 23 non-diabetic patients received steroid therapy, with a pain improvement rate within 3 days of 70 and 56%, respectively, though no statistically significant difference was found (Table 2).

**Table 2:** Clinical characteristics of mononeuritis ophthalmoplegia by diabetes.

Clinical features	DM (43)	Non-DM (25)	*p* value
Age (years)	63.4 ± 8.2	57.7 ± 14.7	0.085
Male	30 (69.8)	10 (40)	0.016
Hypertension	28 (65.1)	12 (48)	0.167
History of CAD	11 (25.6)	1 (4)	0.039
History of stroke	6 (14)	4 (16)	1
Nerve involved			0.192
CN III	30 (69.8)	12 (48)	
CN IV	8 (18.6)	7 (28)	
CN VI	5 (11.6)	6 (24)	
Headache or orbital pain	38 (88.4)	22 (88)	1
Duration of headache *[M(P25, P75)]*	1 (1–2)	1 (1–2)	0.602
Positive head MRI (%)	1 (2.3)	1 (4)	1
Presumed patient with PO (%)	38 (88.4)	22 (88)	1
Complete enhanced MRI (%)	30	16	
Positive enhanced MRI (%)	12 (40)	10 (62.5)	0.100
Stenosis of ICA siphon (%)	22 (51.2)	7 (28)	0.063
Sinusitis (%)	33 (76.7)	12 (48)	0.016
Fasting blood glucose	8.5 ± 2.9	4.9 ± 1.5	0.000
HbA1c (%)	8.1 ± 1.5	5.6 ± 1.4	0.000
Hormonal therapy (%)	38 (88.4)	23 (92)	0.635
Improvement in pain within 3 days (%)	30 (70)	14 (56)	0.151
Duration of hospital stay	10.4 ± 3.4	10 ± 2.8	0.593

## Discussion

There are numerous clinical causes of ophthalmoplegia. From a localization perspective, the lesion may be situated in the brainstem, peripheral nerves, neuromuscular junction, or the muscles themselves. The etiological factors are even more complex, including vascular, infectious, immune-mediated, non-specific inflammatory, space-occupying lesions, trauma, and other causes (3, 4, 6–8). Our study specifically focused on patients with isolated oculomotor nerve palsy. While the etiological scope was more restricted in these cases, but there were still many aspects that warrant further investigation and reflection.

Our study included 68 patients with isolated ocular motor nerve palsy, with an average age of 61 years. There were 43 patients with diabetes and 40 with hypertension. The number of patients with isolated third, fourth, and sixth nerve palsies were 42, 15, and 11, respectively. 60 patients experienced headaches or orbital pain to varying degrees. Previous studies have included different populations, but most suggested that ocular muscle paralysis caused by the 4th or 6th cranial nerves was the most common (9–12). Our findings indicated that the fourth nerve was more commonly affected than the sixth, which may be related to the criteria we used. In clinical practice, some patients with diplopia may not exhibit obvious ocular movement abnormalities, making diagnosis more challenging. In such cases, using the presence of vertical diplopia, head tilt toward the side opposite the symptoms, and relatively normal eye movement as criteria can improve the detection rate of fourth nerve palsy.

From the perspective of painful ophthalmoplegia, the etiology was relatively complex. This diagnosis applied to all patients presenting with ophthalmoplegia accompanied by pain at their initial consultation. After excluding obvious secondary causes through clinical symptoms, signs, medical history, laboratory tests, and imaging examinations, the diagnosis of benign painful ophthalmoplegia seemed straightforward for the 60 patients in our study.

In our study, 60 patients preliminarily diagnosed with painful ophthalmoplegia, 19 were ultimately confirmed as THS. 46 patients were clinically diagnosed with MVP, of which 31 had diabetes and were thus diagnosed with diabetic ophthalmoplegia. Notably, these 31 patients also met the diagnostic criteria for MVP.

Currently, aside from THS (13), which had clear diagnostic criteria, there were no universally accepted standards for diagnosing diabetic ophthalmoplegia and MVP. Based on the current understanding of diabetic ophthalmoplegia, MVP, and THS, a patient could potentially fall under two diagnostic categories. Risk factors for MVP included diabetes as well as other vascular disease risk factors (10, 11), while diabetic ophthalmoplegia was thought to result from localized ischemic nerve damage due to chronic hyperglycemia (3, 9). Patients with diabetes who presented with painful ophthalmoplegia and negative MRI findings were often diagnosed with benign THS, making it difficult to distinguish from diabetic ophthalmoplegia (1, 2).

From the perspective of pain, it seems to have limited significance in the differential diagnosis of ophthalmoplegia. The majority of our patients with isolated ophthalmoplegia also experienced pain. In a cohort study involving 109 cases of isolated ophthalmoplegia, 60% (65 patients) had associated pain or headache, indicating that the presence or absence of pain did not necessarily predict the underlying cause of the ophthalmoplegia (10).

In a retrospective analysis by Daniel et al. (14) of 65 patients over the age of 50 diagnosed with ischemic ophthalmoplegia, 46 patients (70.8%) had either diabetes or impaired glucose tolerance, with 53.8% of them experiencing pain. Another study focused on isolated MVP reported 92 episodes of ophthalmoplegia, with pain presented in 57 episodes (62%) (11). Both diabetic and non-diabetic patients commonly experienced pain. Kaushik et al. (15) studied 35 patients with diabetic oculomotor nerve palsy, finding that 77% had associated headache and 48.5% had orbital pain. Additionally, a high-resolution MRI study involving 10 patients with diabetic ophthalmoplegia revealed that all patients with third nerve palsy also had headache symptoms (16).

These studies suggested that the presence of pain is relatively common in patients with diabetic ophthalmoplegia, and that headache may not be particularly helpful for clinicians in differentiating the underlying etiology of ophthalmoplegia.

Different studies have explored the incidence, etiology, treatment outcomes, and other characteristics of ophthalmoplegia from various perspectives. From the perspective of diabetes, ophthalmoplegia was a relatively rare complication of diabetes, most commonly occurring in elderly patients with long-standing diabetes. The incidence of cranial nerve palsy in diabetic patients was 10 times higher than in non-diabetic patients (3). A large-scale study (9) on diabetes reported an incidence of 0.32% for diabetic ophthalmoplegia. Another study (3) showed that the incidence of ophthalmoplegia in all diabetic patients was 0.4%, with isolated oculomotor nerve palsy accounting for 88.9%. Consistent with our findings, isolated third nerve palsy was the most prevalent, representing 59.3% of cases. However, in previous studies, not all patients underwent brain MRI or orbital MRI examinations, so the exact incidence rate requires further research to be clarified.

The exact etiology of diabetes-related cranial nerve palsy remained uncertain, but it was generally believed to be associated with atherosclerotic changes in small blood vessels. Persistent hyperglycemia leaded to metabolic abnormalities and microvascular damage, resulting in ischemia and hypoxia of the nerves that control eye movement. Previous pathological studies suggested that ischemic nerve infarction was present in patients with diabetic oculomotor nerve palsy, with the disruption of microcirculation considered a possible cause (3).

Contrast enhancement MRI in patients with MVP or DO showed thickening and enhancement of the ipsilateral ocular motor nerve (5, 17). However, whether this nerve enhancement indicated inflammation and whether corticosteroid treatment was necessary remains controversial. Recent high-resolution magnetic resonance imaging (MRI) studies on diabetic ophthalmoplegia have provided new insights. A French study (16) selected 10 patients with diabetic ophthalmoplegia. Among them, 8 cases showed local high signal and enhancement of the affected nerve, extending from the cavernous sinus to the orbit. All patients with third nerve palsy had headaches, accompanied by nerve thickening. The results suggested that ischemia and inflammation were both involved in the pathophysiological processes of neuropathy in type 2 diabetes.

From a differential diagnosis perspective, while we ultimately diagnosed 31 patients with diabetic ophthalmoplegia, this did not necessarily mean the diagnostic process was perfectly concluded. A study aimed at confirming the etiology of painful ophthalmoplegia through long-term observation followed 10 patients with diabetic ophthalmoplegia. One of these patients was eventually diagnosed with cavernous sinus B-cell lymphoma due to disease progression during follow-up (18). For patients whose ophthalmoplegia worsens, who developed new neurological symptoms or signs, or who did not recover within 3 months, a more comprehensive evaluation, including neuroimaging, was necessary. Studies showed that a small proportion of patients initially diagnosed with diabetic ophthalmoplegia were later found to have other conditions during follow-up (19).

From the perspective of microvascular ophthalmoplegia, diabetes is one of the major risk factors, with other contributing factors including hypertension, coronary atherosclerotic heart disease, hyperlipidemia, smoking, and alcohol consumption. Although previous pathological case reports, all involving the third cranial nerve, have been limited to three instances, they suggest ischemic demyelination linked to micro-arteriosclerotic changes in the vasculature surrounding the nerve (11). These findings highlight the potential role of microvascular disease in the development of ophthalmoplegia, particularly in patients with multiple vascular risk factors.

Firstly, the pathological findings observed may not accurately reflect the condition of the oculomotor nerve at the time of disease onset. Secondly, studies have shown that patients who did not fall under the category of MVP may still present with vascular risk factors. Thirdly, it is clinically challenging to directly confirm that ischemia caused ocular motor nerve damage. Given that diabetes was a well-established condition in clinical practice, our research group believed that patients with coexisting diabetes should be directly diagnosed with diabetic ophthalmoplegia.

If we eliminate the diagnosis of MVP, it becomes challenging to diagnose patients who have normal routine test, no accompanying headache, and no diabetes. For this group of patients, we proposed diagnosing them with idiopathic ophthalmoplegia and recommending follow-up to determine whether any secondary causes emerge over time. This approach would ensure more accurate long-term diagnosis and management of these cases.

Since the release of the International Classification of Headache Disorders, 3rd edition (beta version), there has been ongoing debate regarding the differential diagnosis between diabetic ophthalmoplegia and THS (1). The latest diagnostic criteria for THS require abnormal findings on MRI or biopsy. Zhang et al. (2) have questioned the validity of these criteria, as some patients cannot be clinically distinguished from THS. In recent years, some scholars have proposed the concept of benign THS for patients with normal MRI findings, suggesting that such cases should still be diagnosed as THS despite the absence of detectable abnormalities on imaging (20).

A study (21) of 58 cases of painful ophthalmoplegia included 26 patients (44.8%) with diabetic ophthalmoplegia and 27 patients (46.6%) with benign THS. The involvement of cranial nerves was similar between the two groups. Compared to diabetic ophthalmoplegia, patients with benign THS responded more favorably to corticosteroid treatment, with diplopia resolving more quickly. A closer analysis of the data revealed that the average recovery time was 2 months in the diabetic group and 1.6 months in the benign THS group. This suggested that distinguishing between the two diseases based on recovery time was also challenging, as early clinical differentiation remained difficult.

Additionally, a follow-up study (18) indicated that the time to pain relief was similar between diabetic patients and THS patients, though the recovery time for diabetic ophthalmoplegia was slightly longer than that for THS. In 12 patients with THS who received corticosteroid treatment, the average time to pain relief was 33.8 h, and the average recovery time for ophthalmoplegia was 40.7 days. In contrast, for 10 patients with diabetic ophthalmoplegia, the average time to pain relief was 41.1 h, and the average recovery time for ophthalmoplegia was 62 days.

We conducted analysis based on whether patients had comorbid diabetes, and the results showed no significant differences in key indicators of ophthalmoplegia between the two groups. In the diabetes and non-diabetes groups, 11 and 9 cases, respectively, were clinically diagnosed as THS. Additionally, 38 cases and 23 cases in each group received steroid treatment, with pain improvement rates within 3 days of 70 and 14%, respectively.

For patients presenting for their first visit, it is relatively easy to obtain information on the presence of headache, the type of ophthalmoplegia, medical history, routine laboratory tests, head and orbital MRI findings, and the response to corticosteroid therapy. After ruling out secondary causes, the differential diagnoses available included diabetic ophthalmoplegia, ophthalmoplegic migraine, THS, and benign THS.

Currently, the boundaries between diabetic ophthalmoplegia, MVP, diabetes-associated THS, and benign THS remain unclear. In some cases, the interpretation of enhanced MRI findings of the orbit or cavernous sinus can be ambiguous. This highlights the need for clinical research conducted by specialists from neurology, ophthalmology, and otolaryngology to develop unified definitions, classifications, and diagnostic standards. Establishing clear and actionable guidelines will provide clinicians with a practical framework to accurately diagnose and manage these complex conditions.

Our research group proposed the following recommendations. Firstly, for patients presenting with painful ophthalmoplegia and a history of diabetes, after excluding other potential etiologies, a diagnosis of diabetic ophthalmoplegia should be considered. Secondly, in diabetic patients with painful ophthalmoplegia, if MRI reveals enhancement, THS should be diagnosed. In the absence of MRI findings, idiopathic ophthalmoplegia may be considered. Thirdly, due to the challenges in obtaining pathological evidence for MVP, idiopathic ophthalmoplegia may be used as a provisional diagnosis. Lastly, long-term follow-up is crucial for patients initially diagnosed with benign ophthalmoplegia to detect potential secondary causes that may emerge over time.

Our study has several limitations. First, not all patients underwent enhanced MRI scans of the orbit or cavernous sinus, potentially limiting the number of confirmed cases of THS. Second, as this was a retrospective study, data on ophthalmoplegia were collected from historical records, which may have introduced bias, particularly in the diagnosis of fourth cranial nerve palsy. Third, the lack of long-term follow-up in our patients may have led to limitations in accurately categorizing disease progression and determining final etiological diagnoses.

## Conclusion

Currently, the boundaries between diabetic oculomotor palsy, MVP, diabetes combined with THS, and benign THS remain unclear. There is a need for clinical research involving specialists in neurology, ophthalmology, and otolaryngology to establish standardized definitions, classifications, and diagnostic criteria.

## Data Availability

The original contributions presented in the study are included in the article/supplementary material, further inquiries can be directed to the corresponding author.
